# Making Sense of Science, University, and Industry: Sensemaking Narratives of Finnish and Israeli Scientists

**DOI:** 10.1007/s11024-022-09485-6

**Published:** 2023-01-27

**Authors:** Elina I. Mäkinen, Adi Sapir

**Affiliations:** 1 Faculty of Management and Business, Tampere University, Tampere, Finland; 2 Department of Leadership and Policy in Education, Faculty of Education, University of Haifa, Haifa, Israel

**Keywords:** Academic entrepreneurship, Commercialization of science, Entrepreneurial scientist, Sensemaking narratives

## Abstract

Academic entrepreneurship and the commercialization of science have transformed higher education in recent decades. Although there is ample research on the topic, less is known about how individual scientists experience and perceive the transformation. Drawing on a narratological approach to sensemaking, this study examines how entrepreneurial scientists in Finland and Israel make sense of and narrate the perceived changes in the interface between science, university, and industry. An analysis of 53 semi-structured interviews reveals three sensemaking narratives demonstrating how scientists’ interactions with the industry have engendered perceived shifts in ‘regimes of value’ in universities. These narratives focus on: (1) bi-directional learning between academy and industry; (2) the use of new valuation devices and practices; and (3) changing relationships between scientists and universities. Our findings advance research on academic entrepreneurship by highlighting the coexisting regimes of value and the consequences they have for science, value, and power.

## Introduction

Academic entrepreneurship and commercialization of scientific discoveries have gained wide scholarly attention for decades (e.g., Grimaldi et al. 2011; Rothaermel et al. 2007; Shane 2004; Siegel and Wright 2015). However, it has been argued that we have only a limited understanding of how individual scientists experience their interactions with industry (Eriksson et al. 2021: 11; Miller et al. 2018: 27). To advance the research in this area, we explored the experiences of entrepreneurial scientists—those who seek to commercialize their scientific discoveries—through the lens of sensemaking, which signifies the processes by which individuals and groups interpret and construct meaning to their experiences (Weick 1995). Drawing specifically on a narratological approach to sensemaking (Brown et al. 2008), we examined how these scientists make sense of and narrate the perceived changes in the interface between science, university, and industry.

We analyzed data collected in two countries that, despite national, political, and historical differences, present intriguing similarities in how they perceive entrepreneurial efforts in academia. Finland and Israel are ranked among the leading innovative economies (Bloomberg Global Innovation Index 2021), and their higher education field comprises primarily public universities. Finland ranked eleventh in the IMD World Competitiveness Ranking 2021 (World Competitiveness Ranking 2021), and within Europe, it ranked second after Sweden on the European Innovation Scoreboard (European Commission 2021). Finland has performed particularly well on indicators such as lifelong learning, Patent Cooperation Treaty (PCT) patent applications, and international scientific co-publications (European Commission 2021). The technology industry has been Finland’s most significant export industry. More recently, health tech has become the largest and one of the fastest-growing export segments in its high-tech industry (Healthtech Finland report 2020). Although challenged by the COVID-19 pandemic, the value of Finland’s exports of health technology products continued to grow, rising to €2.43 billion in 2020 and to €2.52 billion in 2021. Finland has 13 public research universities, a National Defense University, and 22 universities of applied science. Finnish universities have a specific mandate to support the transformation of scientific knowledge into profitable businesses and promote the creation of new businesses.

Israel holds a leading position in the OCED R&D Intensity Index (R&D investment as a ratio of GDP) and is a world leader in the number of start-ups per capita (Israel Innovation Authority 2019). In 2019, Israel was ranked 5th among OECD countries in inventors’ PCT submissions relative to population size (Leck et al. 2021). Israel is considered a high-tech powerhouse, the most successful country after the United States in establishing high-tech industries (Wonglimpiyarat 2016). In 2021, high-tech exports exceeded 50% of Israeli exports (Israel Innovation Authority 2022). Israel’s higher education system comprises eight public research universities, a private university, an open university, and 49 academic colleges. Its higher education policy supports technology transfer (TT) and collaborations with industry. Each university has an affiliated TT company. Patent applications by universities’ TT companies comprise about 10% of the total inventive activity of Israeli applicants (Leck et al. 2021).

Our analysis reveals three sensemaking narratives that highlight the entrepreneurial scientists’ perceptions of change regarding *regimes of value*, understood as socially constructed logics of value and exchange (Appadurai 1986). These sensemaking narratives reflect three areas of interest: (1) bi-directional learning between academia and industry, (2) new valuation devices and practices, and (3) changing relationships between scientists and universities. Our study makes a twofold contribution to research on academic entrepreneurship. First, by analyzing the sensemaking narratives of entrepreneurial scientists, we tap into their retrospective interpretations of various entrepreneurship experiences and elucidate narratives on topics that have received little or no attention in previous research. Second, we highlight a common thread across the narratives that concerns a sense of change regarding *regimes of value* (Appadurai 1986), which have the power to determine what matters and what is seen as having value in academic science.

We first describe existing research on academic entrepreneurship and then explain how the approach of sensemaking narratives contributes to this research. Next, we explain our data and analytical approach. We present how our findings align with existing research on entrepreneurial motives, roles, and identities. We then demonstrate the three sensemaking narratives that reveal the tensions and compatibilities between scientific-academic and commercial-industrial regimes of value. We conclude by discussing the broader significance of these findings.

## Academic Entrepreneurship

*Academic entrepreneurship* signifies the efforts of higher education institutions and scientists to commercialize scientific discoveries (e.g., Kleinman et al. 2018; Rothaermel et al. 2007; Siegel and Wright 2015). *Commercialization* refers to converting scientific knowledge into intellectual property that can be licensed for profit. More broadly construed, academic entrepreneurship can refer to varied entrepreneurial activities carried out in academia, including entrepreneurship education, the role of managers in the promotion of entrepreneurial activities, and university-industry and university-society relationships (Eriksson et al. 2021). We discuss extant academic entrepreneurship research by focusing on institutional structures and practices and then on scientists’ behaviors and perceptions. Finally, we describe how the theoretical lens of sensemaking advances research on how academic entrepreneurs give meaning to their experiences.

Academic entrepreneurship has been studied from the perspective of institutional change, where universities’ institutional structures and practices are seen as transforming and becoming more heterogeneous (Oliver and Sapir 2017; Siegel and Wright 2015). Indeed, universities have responded to the need to promote the commercialization of academic research by establishing new institutional structures, such as technology transfer offices (TTOs), incubators, science parks, and entrepreneurship education programs that rely on practices aiming to increase interest and know-how in entrepreneurship (e.g., Mowery et al. 2004; Rothaermel et al. 2007; Shane 2004; Siegel and Wright 2015). TTOs’ role is particularly important as they monitor laboratory research and assess new inventions' commercial value (Markman et al. 2005). Even more heterogeneous institutional arrangements are emerging, including start-up accelerators, entrepreneurial and pitching competitions, and university-industry networks (Kleinman et al. 2018; Oliver and Sapir 2017; Siegel and Wright 2015).

Scholars have also argued for the importance of a more micro-level perspective on academic entrepreneurship and have thus addressed scientists’ behaviors and perceptions (Balven et al. 2018; Hmieleski and Powell 2018; Neves and Brito 2020). Scientists differ greatly in how they perceive industry engagement and its potential benefits, even in entrepreneurial universities. Some recognize synergies between industry and academia, and others view them in opposition (Colyvas 2007; Novotny 2017; Owen-Smith and Powell 2001). However, as academia and industry become more interconnected, understandings and values concerning academic research will likely be affected (e.g., Etzkowitz 1998; Kleinman and Osley-Thomas 2014; Kleinman et al. 2018; Slaughter et al. 2004). Studies have shown, for instance, how these changes activate efforts to demarcate academia and industry in their respective work practices (e.g., Ylijoki 2003; Johnson 2018).

Relating to the micro-level perspective, the ongoing transformation in the relationship between academia and industry also affects academics’ professional identities (e.g., Beck and Young 2005; Meek and Wood 2016). Some scholars have argued that academic entrepreneurship challenges or even threatens academic identities (e.g., Meek and Wood 2016; Ylijoki 2005), whereas others note that scientists can have many identities with differing values and behaviors (e.g., Karhunen et al. 2017; Mäkinen and Esko 2022). Nevertheless, engaging in entrepreneurial activity often leads to the need to negotiate professional identities through various identity strategies or by developing hybrid identities (e.g., Jain et al. 2009; Karhunen et al. 2017; Lam 2010; Shi et al. 2021).

While academic entrepreneurship has been studied from the perspectives of institutional structures and practices and scientists’ behaviors and perceptions, we have a limited understanding of how individual scientists experience their engagements and interactions with industry and how they make sense of these experiences (Eriksson et al. 2021: 11; Miller et al. 2018: 27). Next, we describe the theoretical lens of sensemaking narratives, which we use in the present study to explore how scientists ascribe meaning to their experiences with industry.

## Sensemaking Narratives

*Sensemaking* can be defined as “the ongoing retrospective development of plausible images that rationalize what people are doing” (Weick 2008: 1403). Sensemaking is a social process that occurs when individuals and groups engage in processes of interpretation and meaning-making that influence behavior and inform action. Sensemaking is also related to identity construction. Individuals continually make sense of their identity when they give meaning to situations and experiences (Weick 1995). While sensemaking is an everyday, ongoing process, it is especially prevalent in novel or unexpected situations in which “the current state of the world is perceived to be different from the expected state of the world, or when there is no obvious way to engage the world” (Weick et al. 2005: 409). In such contexts, individuals and organizations engage in sensemaking to confer meaning to unfamiliar, unclear, and ambiguous situations. A critical approach to sensemaking examines how dimensions of power, politics, and discourse influence sensemaking processes (Maitlis and Christianson 2014). Scholars have stressed the contested nature of competing sensemaking perspectives, the privileges of certain interpretations and meanings over others in particular organizational contexts, and the relationship between individual sensemaking and broader societal systems of meaning, discourse, and power (e.g., Mikkelsen and Wåhlin 2020; Mills et al. 2010).

A related stream of research concerns the discursive nature of sensemaking. Our focus is on the literature that explores *narrative sensemaking*, that is, the use of narratives to construct meaning from personal or organizational experiences. Narratives, understood as “accounts of value-laden symbolic actions embedded in words and incorporating sequence, time, and place” (Brown et al. 2005: 313), are discursive practices by which we organize our experiences and memories and try to understand those of others (Bruner 1991; Czarniawska 2004). Narratives are versions of reality that reflect unfolding accounts of events or actions over time and are influenced and shaped by the broader narratives of the societies in which individuals are affiliated (Rhodes and Brown 2005). *Stories* can be used interchangeably to narratives (Brown et al. 2005; Brown et al. 2008) or as a particular form of narrative “with simple but resonant plots and characters, involving narrative skills, entailing risk, and aiming to entertain, persuade, and win over” (Gabriel 2000: 10).

Scholars have suggested that sensemaking is, in essence, a narrative process. In his landmark study, *Sensemaking in Organizations*, Weick (1995: 129) argued that stories are critical for sensemaking:

First, stories aid comprehension because they integrate that which is known about an event with that which is conjectural. Second, stories suggest a casual order for events that originally are perceived as unrelated and akin to a list. Third, stories enable people to talk about absent things and to connect them with present things in the interest of meaning. Fourth, stories are mnemonics that enable people to reconstruct earlier complex events. Fifth, stories can guide action before routines are formulated. Sixth, stories enable people to build a database of experience from which they can infer how things work. And seventh, stories transmit and reinforce third-order controls by conveying shared values and meaning.

Studies on narrative sensemaking are concerned with how narratives work as processes of meaning production. Narratives have been considered sensemaking vehicles, lending structure and coherence and memorable and evocative means for constructing shared and divergent understandings (Beigi et al. 2019; Brown et al. 2008; Rhodes and Brown 2005).

Only a few authors have drawn on the theoretical lens of sensemaking to examine how academic entrepreneurs give meaning to their experiences (e.g., Brennan and McGowan 2006). In a series of studies, Moilanen, Montonen, and Eriksson analyzed how Finnish scientists make sense of commercialization activities and academic entrepreneurship in an academic environment with its distinct culture and power hierarchies. Moilanen et al. (2021) delineated the tensions emerging from the juxtaposition of the dominant discourse of academic research and the growing discourse of commercialization that guide the sensemaking of scientists: Whereas senior scientists are less inclined to pursue commercialization, junior scientists aim to hybridize the two discourses. Moilanen, Montonen, and Eriksson (2018) showed how university commercialization systems have failed to provide key resources for scientists’ sensemaking, specifically for creating an academic entrepreneur identity and supporting commercialization activities. Montonen, Eriksson, and Aromaa (2018) explored the sensemaking story of a health scientist and showed how he constructed a self-identity as an in-betweener, in which science and business combine in novel ways, challenging academic norms and expectations.

In this study, we adopt a sensemaking narrative perspective to critically examine how Finnish and Israeli scientists narrate change in academia and assign meaning to their experiences at the crossroads of science, academia, and industry.

## Data and Method

This study is based on 53 semi-structured interviews conducted in Finland and Israel with entrepreneurial scientists at different career levels and working in different research fields. For this study, we define *entrepreneurial scientists* as those who engage in research commercialization and collaborations with industry. In Finland, one author conducted 26 interviews between 2018 and 2020, each lasting between 45 and 90 min; all interviews were recorded and fully transcribed. Among the Finnish interviewees, 16 were male, and 10 were female. Seventeen were from the life sciences, seven from health technology and engineering, and two from physics. Regarding participants’ career stage, 12 interviewees were research group leaders or professors, 12 were senior or postdoctoral researchers, and two were doctoral candidates. The interview protocol included questions such as how the informants had made discoveries that yielded commercial potential, how they experienced their interactions with the industry, and how their work had transformed because of these interactions. The analysis presents representative quotations from Finnish scientists by codes F1–F26.

In Israel, one author conducted 27 interviews, each lasting between 60 and 120 min, between 2009 and 2011; all interviews were recorded and fully transcribed. Twenty-two of the Israeli interviewees were male, and five were female. Seventeen interviewees were from the life sciences faculties, one from physics, one from mathematics, and the remainder from computer science and engineering faculties. One interviewee was a doctoral candidate, and the remainder were faculty members. The interview protocol included questions addressing their motives for turning to commercialization, their experiences working with the industry and other stakeholders, and the consequences of their relationships with the industry on their research and academic status. The analysis cites representative quotations from Israeli scientists by codes I1–I27. Table 1 summarizes the characteristics of the interviewees.

**Table 1:**
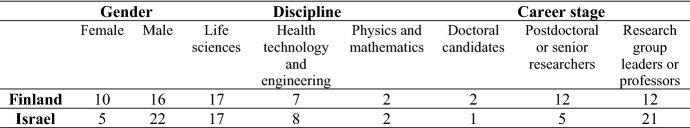
Characteristics of the interviewees

The data were analyzed using narrative analysis. We used the categorical-content approach described by Lieblich et al. (1998) to focus on the content of parts of the narrative linked to the phenomena under analysis. In the first analytical stage, the two authors analyzed the interview data, each focusing on their home country’s interviews with the local scientists. We analyzed all the data in search of sensemaking narratives that reappeared across interview transcripts. These narratives were then translated into English from the Finnish and Hebrew transcripts. In the second data analysis stage, we read the sensemaking narratives that were extracted and translated from the interviews. We ascertained the following themes that appeared in both the Finnish and Israeli accounts: personal stories of how and why the interviewees became entrepreneurially oriented; how they experienced the shifts between basic, applied, and commercial research; learning from and with industry; demonstrating value through new tools and strategies; and rethinking their relationships with the university. For each theme, we collected examples of sensemaking narratives across the interview corpus that we discussed as a team, enabling us to develop a shared understanding of the meanings of these themes. To our surprise, the themes were evident in the accounts of both Finnish and Israeli scientists, with no theme unique to one country.

In the third data analysis stage, we assessed the initial themes against extant research on academic entrepreneurship. To advance research on less-studied issues, we focused on the interviewees’ sensemaking narratives that shed light on how academic entrepreneurship created new experiences, values, and relationships in universities. We began to reanalyze the original themes, this time focusing our attention on three sensemaking narratives: (1) bi-directional learning between academia and industry, (2) new valuation devices and practices, and (3) changing relationships between scientists and universities. We continued to identify sensemaking narratives reflective of *becoming an entrepreneurial scientist*, providing support for the narratives our informants shared and confirming prior findings.

## Empirical Findings: Sensemaking Narratives in Changing Regimes of Values

Three sensemaking narratives emerged from our analysis: a narrative about the bi-directional learning between academia and industry, a narrative concerning the emergence of new valuation devices and practices, and a narrative addressing the changing relationships between scientists and universities. A common thread across these narratives was a perception of change concerning *regimes of value* (Appadurai 1986) produced and reproduced in social interactions embedded in power relations. The narratives highlight assumptions and practices of defining value between scientific-academic and commercial-industrial regimes. The scientists’ accounts reveal the tensions and compatibilities between the regimes of value, their experiences of navigating between ways of framing value, and the implications of new assumptions concerning the concept of value.

Before addressing these narratives, we discuss our findings of scientists’ experiences of academic entrepreneurship, which largely align with studies on how these entrepreneurs construct their identities, explain their motives, and cope with contradictions and tensions embedded in academic entrepreneurship (e.g., Lam 2010; Miller et al. 2018). We then present the sensemaking narratives that facilitate a deeper understanding of the lived experiences and perceptions of change in the context of different regimes of value. Table 2 maps the study’s main findings: scientists’ experiences of becoming academic entrepreneurs and the three sensemaking narratives.

**Table 2:**
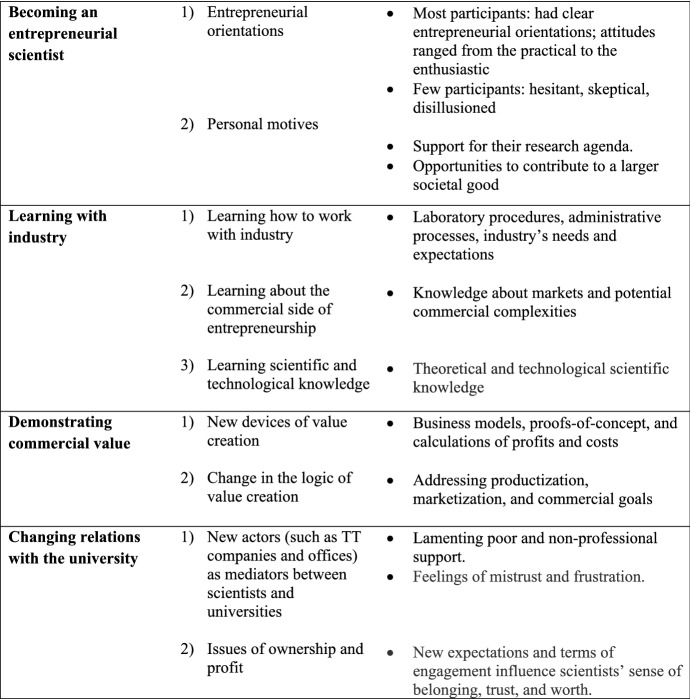
Sensemaking narratives

### Becoming an Entrepreneurial Scientist

Aligning with prior research (e.g., Lam 2010; Miller et al. 2018), most interviewees expressed clear entrepreneurial orientations, readily seeking industry engagement and market opportunities. Their attitudes ranged from the practical to the enthusiastic, reflecting a change in their role identity, evolving from an academic scientist to a hybrid professional identity (Jain et al. 2009; Mäkinen and Esko 2022). For example, a Finnish senior researcher in bioenvironmental engineering reflected on the misguided expectations among academics by claiming:

You are lost if you just stay in the corner of your lab thinking, “Wow, I have this great idea; everyone must want it,” when in fact, nobody wants to hear about it. When you interact with the outside world, you learn what the real needs are and can react to them [F1].

Other scientists expressed an enthusiastic approach, embracing entrepreneurial thinking, norms, and expectations. An Israeli biochemist succinctly described this entrepreneurial mindset:

I find this interface [between academia and industry] fascinating. Because you really want to know if there is something real in what you are doing. And if you don’t have that sparkle in your eyes, it will go nowhere. You must be driven not only by scientific curiosity, but also by the desire to say: “I want to make a drug out of this. I want to make a device. I want this to be the next diagnosis for diseases X, Y, and Z” [I8].

However, some interviewees found their encounters with business and industry overwhelming, challenging, and disappointing (see Moilanen et al. 2021; Shi et al. 2021). These scientists were hesitant and skeptical about their ability to “do entrepreneurship.” As this Finnish professor in bioinformatics explained:

The whole process relies on considerable human interaction, much more than in my usual sandbox. In my sandbox, you have an idea––with experimentation, with facts, with numbers––and once you consolidate your nice idea, you present it to the community and say, “Guys, what do you think?” And then, you collect the feedback. However, in this commercialization business, this does not work. [F5]

Some interviewees expressed disillusionment and frustration following their experiences in entrepreneurship, specifically concerning the normative tensions between the domains of academic science and the market (Jain et al. 2009; Oliver and Sapir 2017). An Israeli scientist in chemical engineering reflected on what she perceived as her naivety in this regard:

When I set out, maybe I was naïve, but I thought about it in bombastic, or maybe altruistic, terms. Something like: “Wow! We were able to invent something that will be cheap [laughs], and it will be possible to use it on a wider scale.” Terribly naïve. The product’s pricing will be determined by considering the profit function and not by any other criterion, such as how to make it easier for physicians to use. And if they do consider this point, it would only be to increase the profit potential. [I2]

Concerning motives for engaging in entrepreneurship, our findings support prior research showing that scientists emphasize the importance of other considerations beyond monetary gains. Two main factors appeared to motivate the commercialization path: support for their research agenda (Lam 2010; Hmieleski and Powell 2018; Rothaermel et al. 2007) and opportunities to contribute to a larger societal good (Jain et al. 2009). Some interviewees described how their scientific work benefited from collaborations with industry, as summarized by an Israeli physicist specializing in optoelectronics:

Projects of this kind make it possible to financially support research activity. So, the financial issue is one issue. Second […] The exposure to your work and your research is more significant if you also go through the path of commercialization. And scientific exposure is basically everything a researcher is interested in […] Third point […] It’s hard to enter a new field because it’s hard to set up the infrastructure. So, this can be a way to establish the infrastructure of a new field, and this is how the researcher can grow in new directions that he had not previously engaged in. [I3]

Regarding contributing to society and the public good, scientists considered commercialization a means to apply scientific research and produce practical solutions to a wide range of problems. As stressed by a Finnish microbiologist, entrepreneurship enabled him to overcome an inherent weakness in academic research:

I have always wanted to do something useful for society because, nowadays, science is mostly a very expensive hobby. […] You feed your own curiosity, and the government is funding it, but there is very little practical output; nowadays, much research leads to nothing. It always was so, but nowadays, there is much more of that. […] And when you have something in hand that seems useful, you of course want to push it further so that people would benefit from it. [F24]

As suggested by previous research (e.g., Lam 2010; Jain et al. 2009; Mäkinen and Esko 2022; Miller et al. 2018), our findings show that many of the Finnish and Israeli interviewees saw value in engaging in commercial efforts, and some even constructed a hybrid professional identity linking academia and entrepreneurship. These scientists described a complementary relationship between academic science and industry in which collaborations with industry enabled them to further their research objectives and contribute to society. On the other hand, some expressed discomfort and disillusionment following their experiences in entrepreneurship.

Next, we discuss the complexities of academic entrepreneurship by focusing on the previously identified three sensemaking narratives that transpire at the margins of the discourse and practice of *academic entrepreneurship* and provide a more nuanced understanding of how entrepreneurial scientists experience and narrate a broader shift in regimes of value (Appadurai 1986).

### Learning with Industry

Academic studies and policy measures concerned with academic entrepreneurship focus on knowledge flows from universities to business and industry. *Technology transfer*, for instance, relates to the transfer of science-based inventions from academia to industry (Kochenkova et al. 2016; Siegel and Wright 2015). A vast literature has addressed the channels, challenges, and barriers concerned with this knowledge transfer process (see Cunningham and O’Reilly 2018). Similarly, the broader discourse of academic entrepreneurship considers entrepreneurial activities as those emanating from scientific innovations developed by academic scientists (Grimaldi et al. 2011; Rothaermel et al. 2007). Thus, the literature relies on the assumption of single-directional knowledge flowing from universities to industry. Few studies have demonstrated how academic entrepreneurship benefits academic research and productivity (e.g., Bikard et al. 2019; Azoulay et al. 2009). In a recent study based on panel data on the total population of academic scientists at a research university, Fini et al. (2022) argued that entrepreneurship shifts scientists’ attention toward new fields of inquiry as it propels them to explore new bodies of knowledge and redeploy concepts and frameworks from the technology domain into the scientific domain.

The present study perceives technology transfer as a bi-directional knowledge transfer process. Our interviewees described how becoming an entrepreneur is a learning process involving various types of learning. First, scientists learned how to work with industry. This new learning began with laboratory procedures and administrative processes and proceeded with acquiring an understanding of industry’s expectations, needs, capabilities, and limitations. A Finnish biochemist involved in commercializing a diagnostic tool described her first-time experience in commercialization:

I’m always learning that to make something commercial as a product, you need to go through many administrative hurdles, [such as seeking] accreditation of the [diagnostic] test. [This process] makes it not research-based but really orients it to the clinical lab. For people to accept it as a clinical test, you need to present convincing data. So, [it needs to be] thoroughly checked and proven. And how does one accomplish this? It’s an entirely new thing for me. It’s not pure science anymore; it’s more statistics and more technical work. Plus, paperwork. To be convincing, you need to submit a pile of papers proving that what you are doing is real. And this, proving that something is real, takes up much of my time. So, I’m learning and consulting with people. [F20]

Transitioning from academic science to industrial science involves a new set of demands regarding laboratory practices, data, and bureaucracy, as well as a change of mindset. In her sensemaking narrative, the scientist explained that moving into this unfamiliar ground (“it’s not pure science anymore”), she encountered new scientific evidence and credibility demands. In this new landscape, she had to learn how to prove that her work was “a real thing.”

A senior Israeli scientist involved in biomedical research with extensive experience in working with industry was determined from an early stage in her career to learn “how things are done in industry” so she could further develop her entrepreneurial projects:

It’s not just about doing the basic research, such as understanding mechanisms etc. You also have to think about how your research will lead to the insights and tools that an industrial company would look at them and say: “This is something I can take and promote, and I don’t need much resources for that; it’s simple; it’s genius.” That’s what should be on your mind. You should also know what industry expects from you. They want to see neat and organized notebooks and good laboratory practice. There are a lot of things in this manner, but you learn these things. You learn how to work with them. […] For example, my invention can solve many cardiovascular diseases in a way that is both effective and solves a particular problem in the cheapest way, making it a more accessible and feasible solution. [I9]

For this scientist, academic entrepreneurship was a learning journey in which she acquired new ways of thinking about collaborations with industry and about her own research. This journey involved significant changes in scientific work, such as learning to accommodate new demands and work standards (“You learn how to work with them”) and managing scientific work according to a new logic of commercialization. Entrepreneurship was then perceived as looking at research from a different perspective––the perspective of feasibility, simplicity, and resource efficiency.

A related dimension of learning concerns learning about the commercial side of entrepreneurship: Feedback from industrial partners can provide a vital learning resource about markets and potential commercial complexities. A Finnish doctoral candidate in microbiology explained:

I can give you an example in our case […] of how we’re developing a treatment for antibiotic-resistant bacteria. […] We had developed a treatment, and we knew that we needed more data, and we needed to advance further into animal models and then into clinical models––clinical studies. But we thought, “Okay, we have developed this treatment for these highly resistant infections. Then the rest will follow.” Still, one of the questions that rose quite quickly was that antibiotic resistance is such a big problem that it has significant implications on the market. […] It gets more difficult to find even noble candidates [for development], but also the rapid resistance [to antibiotics] has led to a couple of cases where companies have spent hundreds of millions to develop a new treatment. They received the *drug of last resort* label, which means that you can only supply it to exceptional cases. Basically, this means that the companies went bankrupt almost immediately after they invested all this money and effort into the development, causing major shockwaves in the market. Those are the things that we, of course, knew nothing about as researchers. [F22]

In this sensemaking narrative, the market is portrayed as an unknown territory to academic researchers in which initial, naïve expectations are met with industrial partners’ practical questions and market expertise. Scientists learned that *antibiotic resistance* could concurrently be a basic science question with practical applications, a scientific discovery with promising commercial potential, and a substantial commercial risk in the pharmaceutical market. Disaster stories about commercial failures and bankruptcies are an important tool for making sense of these tensions and uncertainties.

Finally, interviewees discussed their experience learning scientific and technological knowledge from their industrial partners. This view was dominant among scientists from engineering faculties involved in research collaborations with high-tech companies. Industry was described as a site of knowledge production and innovation, as a senior Israeli scientist specializing in optical communication argued:

Often, researchers actually learn from industry because industrial companies are exposed to other companies. Also, with all due respect to academia, there are intelligent people in industry who have already made more profound progress in specific areas. This can be a fruitful teaching resource for us at the level of theory and research. This is particularly true in Israel, where our strength is in quality and innovation, not quantity. For example, the company I’m involved with is doing things that maybe 2-3 other companies worldwide do. So my side, the academic side, can learn from that. ]I6[

The understanding that expert scientific knowledge resides beyond the walls of academic institutions and that academic scientists can learn from industrial scientists is far from an obvious assumption among academics (“with all due respect to academia”). Nevertheless, this idea seemed to be gaining traction among scientists from engineering faculties. According to an Israeli scientist working on nanoscale information technologies, new relations of knowledge exchange were created in these fields between academia and industry:

In the high-tech industry, there are researchers at an excellent academic level. Take Encryption, for example. I’m sure Check Point^1^ has algorithms as good as ours. Sometimes we bring a good idea, and they bring a good idea […] You come to scientific conferences today and see many articles presented from industry. Most large industrial companies own their own research institutes. [I21]

In these sensemaking narratives, academic entrepreneurship emerged as a space for learning in which expertise resided on both sides of university-industry collaborations, and new types of knowledge and work practices were valued. In these interactions, scientists acquired new insights into commercialization processes, industrial standards of scientific work, and the conditions, characteristics, and complexities of different markets. In many cases, these insights influenced their research agenda and laboratory work, with new thinking about efficacy, feasibility, and market demands producing subtle changes in scientific work. Furthermore, scientists from engineering faculties were adamant that in their collaboration with industry, they gained new scientific knowledge––both theoretical and technological––that affects the practice and content of academic science. This sense of change in assumptions about value indicates a shift in power relations between the university and industry. When they acknowledge that valuable knowledge—empirical, technological, practical, and even theoretical—resides in industry, academic scientists take a step back from the accepted position of the superiority of academic science over industrial science.

### Demonstrating Commercial Value

When seeking to commercialize their discoveries, scientists have to demonstrate that their innovations carry commercial value. This process of productization and marketization of scientific discoveries is achieved with the help of calculative and narrative devices, such as business models, proofs-of-concept (POCs), and calculations of profits and costs (e.g., Doganova and Eyquem-Renault 2009). These devices differ from the familiar means scientists use to *sell* their science in academia. This sensemaking narrative reveals the pitfalls scientists experience when initiating the commercialization process and learning to rely on new devices of value creation.

A Finnish professor developing software in bioinformatics described the difficulties relating to *productization* and his ability to demonstrate that the innovation had value beyond academia:

The biggest problem we have found in this project was essentially not even the commercialization per se but the productization. Basically, how do you go from a nice idea that has proven to be valid and works nicely in an academic environment, and how do you include all the qualities and characteristics needed to convince someone to purchase this as a product? And so, the big problems were not related to the scientific validity of the system. It was rather what I considered in the beginning as something completely useless: How do you package the whole thing? How is the software––in our case, it’s software––how do you package it? What kind of interface do you present? Does it look appealing? What kind of proof do you have that shows it’s effective beyond the scientific proof? This has been a very painful process. [F5]

Here, the interviewee reflected on the difficulty in switching the logic of value creation from science to industry, as it required him to acknowledge that something that he initially perceived as “useless” was now important. Showcasing scientific validity in the academic environment felt straightforward and natural, and he had the required expertise for it; however, developing evidence for showing that the software was “effective beyond the scientific proofs” and attractive to potential users was described as “painful.” As part of this change in the logic of value creation, entrepreneurial scientists are required to learn how to utilize new calculative and narrative devices, thus necessitating acquiring new skills and expertise (e.g., Doganova and Eyquem-Renault 2009). Among our informants, many reflected on POCs and feasibility studies that were crucial when seeking to demonstrate commercial value. Informants who had more experience with commercialization were able to reflect on specific devices and their ability to demonstrate value. A Finnish professor developing human stem cell-based treatments reflected on POCs as milestones on the path toward successful commercialization.

A substantial proof of concept […] is performing animal testing to prove the preliminary functionality of the product. That is our next step. And for us to do this––authorities talk about ‘safety and efficacy’ testing––for us to do those on the level the authorities require with the animal model would be our next big step, which is an expensive project. We are currently applying for an investment round worth about one million euros to advance that on some level, and that is one of our POCs again. So, it kind of consists of many POCs, the way this project is built. We finish one POC, and then we have the next step, which has its own POC, so it progresses piecemeal. It has to consist of pieces because developing this type of product is a 10-year project. […] Probably the biggest challenge is the financial challenge; the next step is always more expensive than the previous one. When we move toward a clinical treatment product, the biggest challenge is its continuity and securing funding for its continuity. [F15]

This account shows how the informant perceived the aim to demonstrate commercial value as an extensive process comprising many costly POCs. As the team was developing a treatment for patients, they had to demonstrate safety and efficacy testing in animal and human studies at various stages. When “the next step is always more expensive than the previous one,” the team had to secure funding for years forward to successfully demonstrate commercial value. The small steps and the burden of guaranteeing funding created a sense of uncertainty. A senior Israeli scientist in electrical engineering who had registered several patents and experience in research collaborations with industry reflected on this uncertainty by describing how at any stage in the process, the devices for demonstrating commercial value could fail:

They asked for materials, and then they asked for a small project, and then a feasibility study and another feasibility study, and demonstrations and discussions, and meetings, and that […] takes a lot of time and energy, also mental energy. And it can take a few months of cumulative work […]. Even when they provide funds, it certainly does not cover the actual costs and its full price, both in our direct work and also, above all, in that our attention is no longer solely directed to that pure, innocent, and beautiful science, but to satisfying concrete requirements to improve the prospect of a positive evaluation of things. […] The problem begins to worsen when there seems to be a fantastic opportunity, and if this contact bears fruit, it will provide such a bonanza that everyone will enjoy––the university and everyone involved––and this thing is dazzling. But for me, all these cases ended with zero results. [I12]

As in the previous account, this informant reflected on the process of demonstrating commercial value as comprising many small moments where success at any one stage facilitates the next. The resources required for each step were significant and difficult to procure and maintain over a long period. Moreover, he described these moments and devices as different from “pure, innocent, and beautiful science.” Instead, the focus was on “satisfying concrete needs,” addressing commercial goals and needs that did not exist in academia. Finally, he captured a critical tension in the sensemaking narrative concerning demonstrating commercial value: Along with the hype regarding potential commercial success is always the prospect of failing at any moment when the commercialization devices do not generate what is needed to progress to the next stage.

This sensemaking narrative concerned with demonstrating commercial value reflected a changed perspective on what had been perceived as valued expertise, thus affecting the balance of power between academia and industry. Entrepreneurial scientists acknowledged that their academic expertise had limitations, as commercialization required productization and marketization devices that were new to them. Moreover, the unpredictability and uncertainty in the commercialization process created a sense that they were not fully in control of the outcomes of their work.

### Changing Relations with the University

The promotion of entrepreneurial science has modified the configuration of relations between the university, the state, and the market (Slaughter et al. 2004). Considering scientists’ experiences and perceptions of the changes in these relationships (e.g., Shinn and Lamy 2006), we present a sensemaking narrative that shows how our informants reflected on their evolving relations with the university. Academic entrepreneurship has led to the emergence of new actors (e.g., technology transfer offices, technology transfer companies, and innovation services) that mediate the relationships between entrepreneurial scientists and their universities in new ways. Our findings indicate significant ambivalence in these changing relations.

First, the entrepreneurial scientists we interviewed were often unclear about who should have the responsibility and the expertise for advancing commercialization. An Israeli scientist shared his frustrations surrounding his experiences in and expectations for working with a TT company.

Basically, the major problem in the university is that they really do not have the know-how to approach the companies […] because they [the TT company] are not within the scope of the scientific front of what’s going on. […] It’s not my business to sell this [product]. It’s the business of the TT company. They should do this work. I developed this. You now go and sell it for me. [I1]

This informant was specific about what he expected from the TT professionals. Selling the innovation was not his business, but he expected other actors to assume that responsibility. A Finnish biomedical scientist similarly reflected on the poor and “amateur-like” support he received from the innovation services.

I felt the innovation services didn’t have the right personnel. It took them six months to tell us that they didn’t want our innovation because they viewed it as too risky. The process was so slow and, in my opinion, amateurish. The novelty search they conducted was pretty poor compared to what I could do with just Googling and gathering background information. Currently, the innovation services have more personnel, but I don’t know what they’re like. Many group leaders I have talked with say they don’t trust the innovation services with their own discoveries. [F2]

Second, in this sensemaking narrative, our informants reported instances where their commercialization efforts led to new types of negotiations between themselves, the university, and its TTOs. Different from academic research, commercializing science and starting a new company resulted in long and uncomfortable conversations concerning the university’s ownership and profit, as a Finnish scientist recounted:

The big challenge was the first six or seven months, which involved transferring these IP rights. [This required] negotiations between the university and the company. But we founded the company. We registered the company. We wanted to start the company sooner, but we couldn’t start because these discussions were going on, and it took six, seven months. It was hard for me to keep the guys I had employed. That was the challenge. […] It was definitely very long. I wasn’t doing anything, just waiting for the university. [F4]

In addition to profit and ownership issues, negotiations between the scientist and the university occasionally led to differences of opinion about which risks were worth taking. These conversations could also strain the trust between the academic institution and the scientist. An Israeli professor of clinical biochemistry recalled her experiences after a spin-off company based on her invention was founded:

When the time came, and the shares were traded on the stock exchange, the TT company acquired an appetite and said: We want to sell all the shares we received when the company was founded. And we didn’t even have any results of the clinical trials yet. […] I worked hard to set up the company and brought funding for research in my [university] lab, and they just sold all the stocks and gave the money to the university. I did not see a penny because I have my own shares. As the chief scientist, I couldn’t sell my shares because it’s like saying: ‘I don’t think the technology will work.’ And that’s about what they said. Everyone else thought they had made the right decision. However, for me, it was like a slap in the face. […] They rushed to sell because they didn’t want to take a risk, which is acceptable. But I felt it was a vote of no confidence in me. [I14]

As the account shows, the scientist realized that in the entrepreneurial landscape, her relations with the university were realigned. While universities generally provide an environment that facilitates scientists to pursue innovative scientific research and evaluate and reward them for their work, commercialization changes the terms of engagement. As this scientist observed, her scientific reputation and loyalty to the university were disregarded in the face of risk management calculations.

This sensemaking narrative demonstrates how entrepreneurial scientists experience a sense of change when it comes to their relations with the university. New actors, such as TTOs, mediate their relationship with the university and play a role in support, roles, value, profit, and ownership issues. This sensemaking narrative sheds light on how different actors—scientists, the university, and TTOs—hold varying power and preference to decide how value should be judged, creating ambiguity and stress in the relationships.

To summarize, the three sensemaking narratives––bi-directional learning between academia and industry, new valuation devices and practices, and changing relationships between scientists and universities––emphasize the scientists’ perceptions of issues that have altered over the course of their careers, especially in the wake of their engagement with industry. A sense of change in what is seen as the logic of value and exchange appeared in all three narratives. While the sensemaking narratives revealed that the scientific-academic logic has not disappeared and, in many ways, coexists with the commercial-industrial logic, the interviews revealed a widely shared sense that what matters and is seen as having value has transformed.

## Discussion and Conclusion

This study contributed to an area of research that has received limited attention: the personal experiences of scientists who work at the intersection of science, university, and industry (e.g., Brennan and McGowan 2006; Eriksson et al. 2021; Moilanen et al. 2021). Previous research has shown that processes characterizing university-industry interactions have prompted changes in vocabularies, practices, and structures that subtly remake the academic field (e.g., Kleinman and Osley-Thomas 2014; Kleinman et al. 2018; Slaughter et al. 2004). Whereas much of the work concerning individual scientists has focused on professional role identities (e.g., Jain et al. 2009; Karhunen et al. 2017; Mäkinen and Esko 2022), we adopted a narrative sensemaking perspective (Weick 1995) to explore how scientists from Finland and Israel drew on their entrepreneurial journeys when making sense of the interface between science, university, and industry. We were interested in the stories in the margins of the *academic entrepreneurship* discourse, in the grey zones (Eriksson et al. 2021), ambiguities, and contradictions that comprise part of the lived experiences of academic entrepreneurs. We combined the theoretical lens of sensemaking narratives with the theory of *regimes of value* (Appadurai 1986) to examine how scientists narrate their perception of change in the logic of value and exchange in their academic field and how they negotiate coexisting regimes of academic science and industry.

In line with previous research on motivation, roles, and identity (e.g., Lam 2010; Jain et al. 2009; Miller et al. 2018), our analysis revealed that many of the Finnish and Israeli interviewees had adopted an entrepreneurial mindset and saw the value in collaborating with the industry to support their research agenda and contribute to society. However, a smaller group of interviewees expressed their frustration with the notion of academic entrepreneurship. These accounts were typically troubled by the normative tensions between academia and industry and the feeling that the commercialization of scientific discoveries was sometimes overstated.

Our main findings revealed three sensemaking narratives, shedding light on academic entrepreneurs’ experiences that have received scant scholarly attention: (1) bi-directional learning between academia and industry; (2) new valuation devices and practices; and (3) changing relationships between scientists and their universities. These narratives reflect scientists’ perceptions of change regarding regimes of value (Appadurai 1986)––that is, the logics of value and exchange that are constructed in social contexts as part of greater transformations in the academic field.

Addressing bi-directional learning, Finnish and Israeli scientists reflected on their learning experiences during their entrepreneurial journeys. The discourse of academic entrepreneurship relies upon the assumption that knowledge flows in one direction––from the university to industry (Grimaldi et al. 2011; Rothaermel et al. 2007); however, our interviewees argued that to become an entrepreneur, one had to learn and negotiate new forms of knowledge. First, there was the know-how and know-what of research commercialization, which includes new laboratory practices, accreditation procedures, industrial expectations, and the complexities of commercial markets. The interviewees valued this new knowledge as a practical tool for promoting entrepreneurial projects and an opportunity to rethink scientific work through new perspectives and understandings. Second, in the engineering disciplines, scientists acquired scientific and technological knowledge from their industrial partners, acknowledging the value of industrial science in the academic context. We argue that the understanding that knowledge flows in both directions has implications for regimes of value in higher education. Academic entrepreneurship emerges from these narratives as an opportunity to learn and create new scientific knowledge in academic laboratories. Thus, the exchange between academia and industry comprises an exchange of valuable knowledge, benefiting both sides. Sensemaking narratives of technology transfer as a bi-directional knowledge transfer process also indicate a change in the balance of power concerning the value of scientific knowledge. Scientists recognize that valuable knowledge resides in both sides of university-industry relations and reveal a willingness to learn from their industrial partners.

The second sensemaking narrative focused on how the need to demonstrate commercial value involved a movement between regimes of value and their related difficulties and pitfalls. Entrepreneurial scientists were compelled to adapt to new devices and criteria to establish value anchored in short-term, concrete needs related to productization, marketization, and functionality (e.g., Doganova and Eyquem-Renault 2009). These devices presented a sharp contrast with the traditional methods of evaluating scientific validity and credibility, thus revealing a gap in the scientists’ expertise. They lamented the costs of this aspect of research commercialization in terms of financial resources, time, energy, and the hidden costs related to scientific work. The scientists noted that these costs were incurred on the slippery slope from basic science to production, as the dazzling prospects of commercial triumph gradually eroded their original research agenda. Demonstrating commercial value proved to be a risky endeavor with high prospects of failure. Instead of experiencing the new valuation devices and practices from the perspective of bi-directional learning, as in the first sensemaking narrative, entrepreneurial scientists struggled to come to terms with having to deal with new types of risks, uncertainties, and strategies for managing them.

The third sensemaking narrative, concerning the changing relationships between scientists and universities, noted the effects of the perceived changes in regimes of value on feelings, dispositions, and relationships. Finnish and Israeli scientists described the emergence of new actors in the organizational environment of universities, such as TT companies and TT offices. These actors operated as mediators between academia and industry but also between universities and scientists. However, scientists tended to question the expertise of TT professionals in promoting commercial value, with the relationships between the two groups often characterized by mistrust and frustration. Additionally, the new valuation criteria impacted the relationships between scientists and universities. Our findings suggest that scientists have experienced a change in these relationships, which are increasingly defined and negotiated through intellectual property, profit, and ownership, thus affecting the power relations between scientists and universities. The new expectations and terms of engagement clash with the traditional criteria of scientific value (i.e., basic research, publications, and grants). The tensions between these coexisting regimes of value have had an impact on scientists’ sense of belonging, trust, and worth.

Overall, these sensemaking narratives reveal a recurring theme of a perceived broader shift in regimes of value (Appadurai 1986) in academia. Entrepreneurial scientists are compelled to negotiate coexisting regimes of academic science and industry. New assumptions about valuable forms of knowledge and practice shape their everyday experiences as academics and entrepreneurs. Scholars in valuation studies have noted that the setting, procedures, and devices involved in valuation practices all have an impact on their outcomes (Helgesson and Lee 2017; Lamont 2012).

In the sensemaking narratives, scientists reflected on the subtle forms of influence on scientific practice and academic culture. These processes involved tensions between ways of framing value, leading to feelings of uncertainty and ambivalence. Scientists spoke about their struggles to establish the commercial value of their scientific inventions and learn about industrial demands and the intricacies of commercial markets. Considering these demands, they noted their efforts to accommodate their scientific work accordingly. Notably, they discussed the new perspectives, insights, and innovative knowledge emerging from collaborations with industry.

More broadly, value and exchange are linked by the *politics of value* (Appadurai 1986), as valuation involves and reflects power relations. The insight that industry possesses valuable knowledge and practices has implications for the balance of power between academia and industry. The redefinition of relationships between scientists and universities regarding ownership and profit comprises an additional implication. As part of the changes in the regime of value, the value of academic entrepreneurs as academic scientists continues to be negotiated and redefined in accordance with novel forms of value and prestige.

As in all research, our study has limitations. The datasets collected in Finland and Israel differed in some ways (e.g., time of data collection, disciplines, and informants’ academic rank), which could impact our informants’ experiences with the industry. Yet, the commonalities in our analysis regarding scientists’ experiences and interpretations were striking. For example, whereas scientists from engineering faculties proved more open to learning with and from industrial partners than their colleagues from the life sciences faculties, these differences were consistent across the two studied countries. Moreover, while our data were collected at different time points, the central themes in the sensemaking narratives were highly similar. In other words, scientists from both national groups made sense of and narrativized their experiences in similar ways.

## Footnotes

1 Israeli High-tech company.
